# Interactions between CYP3A4 and Dietary Polyphenols

**DOI:** 10.1155/2015/854015

**Published:** 2015-06-09

**Authors:** Loai Basheer, Zohar Kerem

**Affiliations:** Institute of Biochemistry, Food Science and Nutrition, The Robert H. Smith Faculty of Agriculture, Food and Environment, The Hebrew University of Jerusalem, P.O. Box 12, 76100 Rehovot, Israel

## Abstract

The human cytochrome P450 enzymes (P450s) catalyze oxidative reactions of a broad spectrum of substrates and play a critical role in the metabolism of xenobiotics, such as drugs and dietary compounds. CYP3A4 is known to be the main enzyme involved in the metabolism of drugs and most other xenobiotics. Dietary compounds, of which polyphenolics are the most studied, have been shown to interact with CYP3A4 and alter its expression and activity. Traditionally, the liver was considered the prime site of CYP3A-mediated first-pass metabolic extraction, but *in vitro* and *in vivo* studies now suggest that the small intestine can be of equal or even greater importance for the metabolism of polyphenolics and drugs. Recent studies have pointed to the role of gut microbiota in the metabolic fate of polyphenolics in human, suggesting their involvement in the complex interactions between dietary polyphenols and CYP3A4. Last but not least, all the above suggests that coadministration of drugs and foods that are rich in polyphenols is expected to stimulate undesirable clinical consequences. This review focuses on interactions between dietary polyphenols and CYP3A4 as they relate to structural considerations, food-drug interactions, and potential negative consequences of interactions between CYP3A4 and polyphenols.

## 1. Introduction

Cytochrome P450 enzymes (P450s) are responsible for the metabolism of a wide range of endogenous compounds (steroid hormones, lipids, and bile acids), as well as xenobiotics including drugs, environmental pollutants, and dietary products [1–4]. P450 enzymes are widely distributed among the phylogenetic trees [5] and considered as a significant player in the world around us, where life and the earth itself would be visibly different and diminished without cytochrome P450s [6]. A direct impact on humans is mediated especially through our own set of 57 P450s [7]. CYP is an abbreviation for cytochrome P450; the gene family is then indicated by a number following the letters “CYP.” Subfamilies are represented by a letter that is followed by yet another number to indicate the specific gene. For example, for the enzyme CYP3A4, “3” stands for the gene family, “A” for the subfamily, and “4” defines the gene that encodes a specific polypeptide [8].

Among this large family of oxidizing enzymes, CYP3A4 is recognized as the main enzyme involved in the metabolism of drugs in the liver and, no less importantly, in the gut. Hence, potential interactions between promising new drugs and CYP3A4 are assessed starting at the early stages of their development [9–11]. CYP3A4 is most abundant P450 in the human liver, accounting for 30% of the total P450 protein content but is also expressed in the prostate, breast, gut, colon, small intestine, and brain [12–17]. In the small intestine, CYP3A enzymes represent the principle drug-metabolizing system and account for approximately 80% of total P450 content [18–20]. Although the total amount of CYP3A expressed in the human small intestine represents approximately 1% of the amount expressed in the liver [21, 22], substantial drug extraction takes place during the absorption of orally administered drugs [23–26]. Orally administered substrates must pass through enterocytes while they can bypass hepatocytes by remaining in the sinusoidal blood before reaching the systemic circulation. The remarkably lower blood flow to the intestinal mucosa as compared to the liver allows for prolonged exposure to the intestinal metabolizing enzymes and lead to relatively high enterocytic drug concentrations. The predominance of CYP3A4 in human intestine and its high capacity enable it to can act several-fold more efficiently in the intestine than in the liver [20, 27, 28]. Furthermore, the intestine receives not only dietary compounds, but also phase I and II metabolites that have been excreted back into the intestine through the enterohepatic cycle [29, 30]. All these facts indicate the importance of intestinal CYP3A4 activity in the metabolism of dietary constituents. In rodents, the isofrom CYP3A is expressed predominantly in the liver, with only scant expression observed in the intestine [31–33]. The different isoforms and distinct expression levels and patterns for P450s in the intestine between humans and rodents limit the suitability of rodents as a model to predict drug metabolism or oral bioavailability in human [34]. This points the importance of studying the effects of ingested polyphenols and other dietary substrates on the metabolism of intestinal CYP3A4 in humans or in models other than rodents' intestine. The latter include cell cultures, microsomes, and microorganisms that express the specific P450 of interest or a whole array of P450s [35–39].

**Figure 1:**
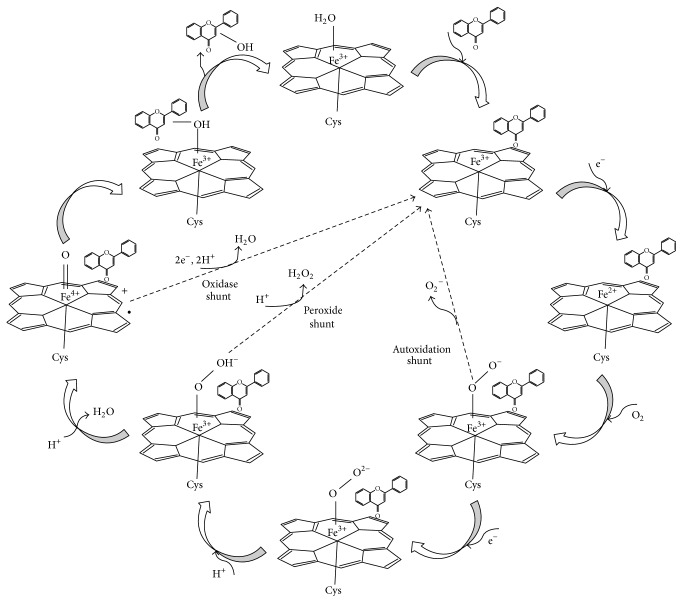
The catalytic cycle of P450s: a flavonoid structure was selected to represent dietary polyphenols.

The active site of a substrate-free cytochrome P450 contains one-heme iron center anchored by the four bonds of the heme group, fifth proximal ligand of the conserved cysteine, and water molecule as the sixth distal ligand [1]. The catalytic mechanisms of P450 enzymes are thoroughly investigated in the literature, as demonstrated in a scheme based on previous publications (Figure 1) [1, 40–42]. Like most other P450 enzymes, CYP3A4 acts as a monooxygenase (e.g., it catalyzes the insertion of one atom of oxygen into an organic substrate while another oxygen atom is reduced to water) [43]. The substrate chemical characteristics and the preferred position of hydroxyl insertion change from one family of P450 to another [3, 44–46]. P450 enzymes play a major role in phase I metabolism of dietary xenobiotics, including polyphenols, whereby a hydroxyl group is introduced to the molecule. These metabolic products are more water-soluble and become available to phase II enzymes. The latter include UDP-glucuronosyl transferases and sulfotransferases that add to the increased water solubility of the hydroxylated polyphenols, producing glucuronides and sulfates, which are then eliminated from the body [29, 47, 48].

In recent studies, evidence has accumulated to indicate potent interactions between CYP3A4 and edible phytochemicals. These compounds, some of which are abundant in our diet, belong to the large and diverse family of polyphenolics, including flavonoids, phenolic acids, phenolic alcohol, stilbenoids, and lignans [49–53]. It is commonly accepted that the powerful antioxidant activity of polyphenolic compounds is due to their free-radical scavenging capacity and their iron-chelating activity [54–56]. Reviews of the health benefits of polyphenols demonstrate that these compounds have numerous therapeutic effects against several diseases (e.g., atherosclerosis, certain forms of carcinogenic processes, and cardiovascular and neurodegenerative diseases) [57–60]. Among the therapeutic implications of polyphenols on human health, the interactions between polyphenols and cytochrome P450 have been recently reviewed [56, 61–64]. These interactions were highlighted following the increased use of herbal medicines and supplements. As many of the herbs used in these preparations are known to be rich in polyphenolics, their interaction with the major enzyme of presystemic metabolism has attracted significant research attention [56, 65–67]. Since cytochrome P450 enzymes are responsible for the metabolism of a wide range of drugs and polyphenols, which might also change their antimicrobial potential and human toxicity, the simultaneous consumption of drugs, herbals, and plant foods raises concerns. The coadministration of active constituents derived from food or herbs and prescribed drugs may lead to undesirable clinical effects, which may include increased toxicity and/or treatment failure [67–69].

Here, we focus on the interactions of polyphenols with CYP3A4, the major enzyme in the gut and liver metabolism of drugs and xenobiotics. The effects of several subcategories of polyphenols on the expression and activity of CYP3A4 (inhibition or induction) are reviewed (Table 1). Structural and physicochemical considerations that define these interactions are also reviewed.

## 2. CYP3A4 and Food-Drug Interactions

Drug-metabolizing P450s such as CYP3A4 have relaxed selectivity and are able to bind and metabolize a large array of substrates of different size, shapes, and chemical properties, for example, many dietary polyphenols. Crystal structures, biophysical studies, and molecular dynamics have provided important insights into how drug-metabolizing P450s, especially CYP3A4, structurally adapt to a variety of inhibitors and substrates [70]. Indeed, CYP3A4 is involved in the metabolism of over 50% of marketed drugs that undergo metabolic elimination [71]. The high level of CYP3A4 expression in the intestine, as well as its broad substrate specificity may explain the accumulating data regarding its susceptibility to modulation by food constituents [38, 61, 72–75]. Examples of metabolic food-drug interactions involving the modulation of CYP3A4 activity by components from dietary and herbal sources are accumulating, including those of grapefruit with over 85 drugs, for example, cyclosporine and felodipine [27, 76–78], and those of St. John's wort [54, 79, 80], and red wine [38, 75, 81] with cyclosporine. In most of these cases, components in foods, drinks, food additives, and orally administered medicines were shown to inhibit CYP3A4 activity and, as a result, increase the actual dose of the drug that reaches the blood circulation in its active form, which often causes unfavorable and long-lasting interactions and probably fatal toxicity [82, 83]. Continuous exposure to these compounds, especially those that activate the xenobiotic nuclear receptor PXR (pregnane X receptor), may lead, in a feedback fashion, to increased expression of CYP3A4 in the intestine, making the food-drug interaction even more complex during extended periods of use [84–87]. Drug-drug, food-drug, and herb-drug interactions in the liver have been well documented in the literature [72, 88–90]. An intensive CYP3A4-dependent intestinal metabolism of low-absorbed compounds such as most polyphenols might be expected [29, 54, 91–93]. However, to the best of our knowledge the research in this area is limited and additional data are needed.

## 3. Polyphenols

**Figure 2:**
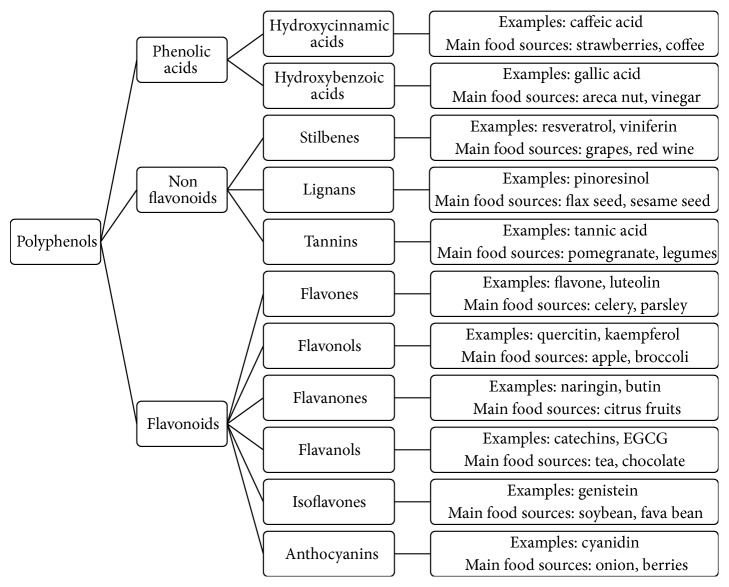
Classification of polyphenols.

As reviewed in other works in this issue, polyphenols constitute a large and diverse family of compounds that is commonly divided into subfamilies that share similar chemistry: flavonoids, flavonols, phenolic acids, phenolic alcohols, stilbenoids, tannins, and lignans (Figure 2). Polyphenols are found in several foods, fruits, vegetables, and herbs [52, 94, 95]. In general, the total intake of polyphenols is approximated at 1 g/individual/day and polyphenols are considered by many to be the major source of antioxidants in our diet [51, 95–97]. However, this estimate varies depending on the type of diet. For example, total polyphenol intake in the Finnish diet is 817–919 mg/individual/day [98]. In the Vietnamese diet, it is 595 mg/individual/day [99], and in the Mediterranean diet, polyphenol intake ranges between 1800 and 3000 mg/individual/day [100]. Still, and due to their low absorption, it has been suggested that their major sites of antioxidant activity are the stomach [101] and the intestine [102]. Whether acting in the gastrointestinal tract or in the liver, the potent antioxidant effects of polyphenols are widely accepted as health promoting [103–105]. Antivira, antibacterial, anti-inflammatory, neuroprotective, and anticarcinogenic effects have also been attributed to polyphenols [106–109]. Medicinal herbs such as St. John's wort (*Hypericum perforatum*), ginseng (*Panax ginseng*), black cohosh (*Actaea racemosa*), echinacea (*Echinacea purpurea*), cranberry (*Vaccinium macrocarpon*), and ginger (*Zingiber officinale*) are rich sources of a vast array of polyphenolic compounds [74, 110–115]. The biochemical mechanisms underlying metabolic herb-drug interaction were well described in a recent review [72]. These herbal sources of polyphenols deserve special attention when the activity of P450s is discussed, due to the dramatic increase in the use of herbal medicines and supplements [65, 66]. Recent surveys suggest that one in three Americans use dietary supplements daily and among cancer patients the rate is much higher [54]. Moreover, medicinal herbs are not inspected by regulatory authorities such as the Food and Drug Administration (FDA) and the European Agency for the Evaluation of Medicinal Products (EMEA) [72]. Indeed, medical doctors as well as pharma professionals should be aware of the many interactions of polyphenolics with drugs and tools should be developed to assess the potential of individual polyphenolics to enter the active sites of P450 enzymes and become substrates, competitive inhibitors, or other types of inhibitors of these enzymes in the intestine and the liver. CYP3A4 should be a major point of focus in studies of the undesirable clinical consequences of the timed use of prescribed drugs and herbs [74].

## 4. Metabolism of Polyphenols by P450s

### 4.1. Metabolism of Polyphenols by P450 Enzymes

The metabolic fate of polyphenols is largely dictated by their chemical structure and depends on several parameters, including their functional groups (i.e., benzene or flavone derivatives), molecular weight, stereostructure, glycosylation, polymerization, and conjugation with other phenolics [97, 116, 117]. Flavonoids, which are the largest subgroup of polyphenols, have been identified as substrates of P450 enzymes [118, 119]. Flavonoids are hydroxylated and/or* o*-demethylated by various hepatic P450 enzymes prior to their elimination [67]. Jančová and coworkers showed that silybin, a flavono-lignan found in silymarin, is metabolized to* o*-demethylated product by CYP2C8 and CYP3A4* in vitro *[120]. Meanwhile it has been reported that flavonoids rich with hydroxyl group such as green tea catechins are fairly water soluble and are not likely to be good substrates for P450 enzymes [121, 122]. This is consistent with findings that have demonstrated the importance of ligand hydrophobicity for interactions with these enzymes [38, 123, 124]. Paradoxically, inhibitory effects of green tea catechins on several P450 enzymes have been reported in* in vivo* trials [125, 126]. Another intensively studied polyphenol is the stilbene *t*-resveratrol (trans-3,4′,5-trihydroxystilbene), a polyphenol found in grape skins and red wine, peanuts, and a limited number of other plants, and its effects on CYP3A4 will be discussed later (Section 5.2.1). It exhibits a high level of membrane permeability and is categorized as a class-II compound in the Biopharmaceutical Classification System (BCS) [127]. *t*-Resveratrol has a low bioavailability (less than 1%) due to the low water solubility (a log*P* of 3.1), and the extensive first-pass metabolism by CYP3A4 in the intestine and in the liver, which extended by the enterohepatic recirculation. Further metabolism leads to the formation of the glucuronide and the sulfate metabolites of *t*-resveratrol [128, 129]. Recently, Singh and Pai reported the success of a systematically optimized nanoparticulate drug delivery system to increase the oral bioavailability of *t*-resveratrol in rats [130]. In a similar context in* in vitro* study, Seljak et al. developed a mixed lipid–mixed surfactant self-microemulsifying drug delivery system (SMEDDS) to improve the biopharmaceutical, pharmacokinetic, and toxicological characteristics of resveratrol, suggesting a way to lower the applied dose of resveratrol, to reduce toxicity while maintaining a sufficient pharmacological response [131].

### 4.2. Involvement of Microbiota in the Metabolism of Polyphenols

There is accumulating evidence to suggest that gut microbiota play a significant role in the metabolism, bioavailability, and bioactivity of dietary polyphenols [132–134]. The involvement of microbiota in the metabolism of these compounds generally starts with the hydrolysis of polymeric, glycosylated and/or esterified polyphenols by brush border and/or microbial enzymes, which is a prerequisite for the absorption and bioactivity of most compounds [134–136]. These biotransformations affect the structural characteristics of polyphenols and may generate metabolites with altered bioactivity profiles [30, 134]. Considering the water soluble green tea catechins, which should be very poor substrates for CYPs, their biotransformation by human gut microbiota could lead to the formation of better CYP substrates, as was demonstrated* in vitro* by Stoupi et al. [137]. Taken together, we suggest that gut microbiota may play a role in the formation of polyphenol-derived metabolites that are more likely to interact with P450 enzymes. The role of intestinal microbiota in the metabolism and bioavailability of dietary polyphenols has been examined [30, 132–136], but, unfortunately, data on the three-way interactions between polyphenols, microbiota and P450s are scarce.

## 5. Modulation of CYP3A4 Activity by Polyphenols

Interactions between polyphenols and CYP3A4 are important due to their potential implications for drug metabolism. These interactions can modulate the activity or expression of the enzyme. Kimura et al. demonstrated inhibitory effects of polyphenols on human CYP3A4 and CYP2C9 activity* in vitro* [73]. These inhibitory effects generally involve the formation of a covalent bond between the polyphenol and the CYP3A4 molecule, which leads to the inactivation of the enzyme, or reversible binding that causes reversible inhibition [138]. In some cases, the inhibition of P450 enzymes by polyphenols may have a chemopreventative effect, due to the potential activation of carcinogens by P450 enzymes within the course of their natural metabolic activity [81, 139–142]. The inhibition of xenobiotic-metabolizing phase I enzymes (i.e., P450 enzymes) could be one target of the chemopreventive effects of naturally occurring polyphenols. Alternatively, it could be the induction of phase II conjugation enzymes, such as UDP-glucuronosyl transferase and glutathione S-transferase, which are responsible for the detoxification of carcinogens [54].

### 5.1. Interactions between Flavonoids and CYP3A4

In large, flavonoids account for about two-thirds of the total intake of dietary polyphenols and phenolic acids account for the remaining one-third [33]. Flavonoids, which are found primarily in fruits, vegetables, and beverages such as tea and wine are bioactive compounds that carry several benefits for human health [142–144]. Flavonoids are known to modulate several P450 enzymes, including CYP1A1, CYP1A2, CYP1B1, CYP2C9, CYP3A4, and CYP3A5 [145, 146]. Hence, their interactions with CYP3A4 are studied in more systems than most other polyphenols and provide evidence for various interactions of polyphenols with this enzyme. There is accumulated evidence that within the family of polyphenols, flavonoids especially can modulate drug metabolism, and in several modes: by altering the expression and/or activity of P450 enzymes, by affecting the P-glycoprotein-mediated cellular efflux of drugs and/or by inhibiting the intestinal glucuronidation of the drug. This evidence indicates that the use of flavonoid-containing dietary supplements concurrent with conventional pharmacotherapeutic regimens should be considered in order to avoid drug-flavonoid interactions [54, 72, 143–146]. In this direction, studies are being conducted to develop methods for evaluating food-drug interactions. For example, Koe and coworkers recently developed a novel multiplex RT-qPCR* in vitro* assay to examine the P450 enzyme-induction properties of herb-derived compounds [147].

#### 5.1.1. Flavonols

The flavonols kaempferol, quercetin, and galangin inhibit CYP3A4-mediated metabolism of xenobiotics* in vitro* [87, 148, 149]. Studies performed* in vivo* have shown conflicting modulation of CYP3A activity by quercetin. Choi et al. reported that oral administration of quercetin to rats led to inhibition of CYP3A, which caused a significant enhancement in the doxorubicin concentration in the plasma. On the contrary, Yu et al. reported an activation of the enzyme that resulted in a reduction in the plasma concentration of cyclosporine in a similar model. The latter observation suggests that this enzyme is not activated by the flavonols, but by their sulfated or glucorunidated products [150, 151]. No* in vivo* inhibition of CYP3A4-mediated metabolism of nifedipine was observed following the ingestion of a high dose of quercetin by others [152]. Interestingly, prolonged exposure to quercetin leads to a significant increase in CYP3A4 mRNA expression levels in cell cultures [87, 153]. We suggest that these findings might be related to the well-established induction of CYP3A4 in response to consumption of St. John's wort extract, which is a rich source of quercetin, in addition to another recognized inducer, the nonphenolic hyperforin [54, 85, 86, 154]. Kaempferol and quercetin have been found to inhibit intestinal UDP-glucuronyl transferase* in vitro* at clinically achievable concentrations, which may lead to an increase in the bioavailability of several drugs [146]. A recent study conducted on rats found that oral administration of morin, a flavonol found in many fruits and herbal medicines, increased the plasma half-life (*t*_1/2_) of febuxostat, a drug used to treat gout 2.5-fold as compared with the control group, leading to significantly higher bioavailability. One suggested mode of action was that morin could be effective in inhibiting CYP1A1, CYP1A2 and CYP3A mediated metabolism of febuxostat [143].

#### 5.1.2. Flavones

The flavones apigenin and chrysin have a marked inhibitory effect on CYP3A4 activity* in vitro*, with IC_50_ values of 0.4 *μ*M and 0.9 *μ*M, respectively. Amentoflavone (a dimer of apigenin) has even a stronger inhibitory effect, with an IC_50_ value of 0.07 *μ*M [73]. Calculations of the lipophilicity of the two compounds provide support for previous suggestions that higher lipophilicity may contribute to stronger binding of the substrate. It is also possible that the larger stereodimensions of the dimer may lead to irreversible binding of the hydroxylation product to the enzyme, thereby achieving inhibition via a suicidal mode of action [12, 155]. A recent study in rats suggests that the coadministration of apigenin would be very useful for improving the bioavailability of paclitaxel in chemotherapeutic applications, due to the inhibitory effects of apigenin on CYP3A and P-glycoprotein, leading to higher concentration of paclitaxel in the plasma [144]. The ability of apigenin to inhibit intestinal UGT activity has also been investigated* in vitro* [146]. In a study designed to reveal structure-activity relationships, flavones possessing more than two hydroxyl groups (e.g., luteolin and diosmetin) were shown to inhibit the biotransformation of midazolam* in vitro*, whereas flavones that do not have hydroxyl groups in their A and B rings (e.g., flavone and tangeretin) stimulated midazolam metabolism [156]. These results may support the activation effect of *α*-naphthoflavone (a flavone with no hydroxyl groups) on CYP3A4 and two other CYP3A enzymes, CYP3A5 and CYP3A7 [11]. In addition, *α*-naphthoflavone represents an interesting case of heterotropic cooperativity in CYP3A4, as it interacts with a peripheral ligand binding site, located at the distal surface of the enzyme and surrounded by the F/F9 and G/G9 loops, resulting in allosteric mechanism [157–161].

#### 5.1.3. Flavonols

Green tea flavonols epigallocatechingallate (EGCG) and epicatechingallate (ECG) inhibit the mutagenic action of aflatoxin B_1_ (AFB_1_) and 1′-hydroxylation of midazolam* in vitro*. Both actions are known to be mediated by CYP3A4 [139]. Inhibitory effects of catechins on CYP3A4 have been reported in several additional* in vitro* and* in vivo* studies, but no specific mode of action has been suggested [73, 121, 125, 126, 162].

#### 5.1.4. Flavanones

The inhibition of CYP3A4 by grapefruit juice is probably the most well-known example of food-drug inhibition [76, 163]. It was suggested that the flavanone naringin, the predominant flavanone in grapefruit, might be responsible for the observed interaction effect [164]. However, naringin appears to be a weak inhibitor of CYP3A4, while its aglycone, naringenin, may be a more potent inhibitor. The IC_50_ value of naringin is 10-fold greater than that of naringenin* in vitro* and this difference is attributed to the lack of a hydroxyl group on ring A of naringin [73, 165]. This is in agreement with the finding of Shimada and coauthors regarding the importance of the hydroxylation of ring A flavones for the inhibition of CYP3A4 [148]. However, the most potent inhibitor of CYP3A4 in grapefruit has been suggested to be bergapten, a furanocoumarin derivative [165], that does not belong to the polyphenol family, but has a relatively similar structure. The inhibitory effects of other furanocoumarins on CYP3A4 activity* in vitro* are also well established [166–168].

#### 5.1.5. Isoflavones

Isoflavones such as genistein and daidzein are found in soybean and hence are very abundant in many processed food products. Isoflavones differ from flavones in the location of their phenyl group. It has been suggested that isoflavones may act as phytoestrogens and they appear potential substrates or inhibitors of P450 enzymes. Conflicting data have been presented in several works describing* in vitro* and* in vivo* studies. For instance, soy isoflavones have been found to inhibit CYP3A4 metabolism [169–171], whereas the administration of genistein resulted in a modest induction of CYP3A enzymes among healthy participants [172, 173].

#### 5.1.6. Anthocyanins

Dreiseitel et al. found that anthocyanins and their aglycones are weak inhibitors of CYP3A4* in vitro* [174]. The IC_50_ values of anthocyanin derivatives ranged from 12.2 to 7,842 *μ*M; whereas ketoconazole, a synthetic CYP3A4 inhibitor that is often used as a reference, has an IC_50_ value of 18.4 nM. Measurement of the IC_50_ values of the different aglycones revealed an inverse relationship between the number of sugar moieties per compound and the ability of anthocyanins to inhibit CYP3A4 [174]. This provides further support for the accumulating data pointing to the importance of lipophilicity for interaction with CYP3A4 [38, 123, 124]. We recently reached a similar conclusion using software to study docking of polyphenols, in which we observed a correlation between the log*P* values of ligands and their docking energies with CYP3A4 (CDOCKER energy expressed in Kcal/mole; Basheer and Kerem, unpublished data).

### 5.2. Interaction between Nonflavonoids and CYP3A4

#### 5.2.1. Stilbenoids

The inhibitory effects of *t*-resveratrol on CYP3A4* in vitro* and* in vivo* are well established, and it has been suggested that resveratrol might act as an irreversible, mechanism-based inactivator of this enzyme [38, 175–179]. This inhibition occurs when a CYP3A4 substrate/inhibitor forms a reactive intermediate at the CYP3A4 active site, leading to enzyme inactivation by modification to the heme or the apoprotein [180, 181]. Chan and Delucchi suggested that an electron-rich unsaturated molecule like resveratrol could be a substrate for CYP3A4 and might, in turn, inactivate CYP3A4 during the course of catalysis [175]. Clinical and rat trials have found that the administration of resveratrol increases the area under the plasma concentration-time curve (AUC) for several drugs [81, 177]. Thus, consuming large amounts of resveratrol could theoretically increase the bioavailability of and risk of toxicity from drugs that undergo extensive first-pass metabolism by CYP3A4 [179].* In vitro* study of the effect of lipophilicity on the interactions of resveratrol derivatives with CYP3A4 revealed that methoxy-stilbenes have lower IC_50_ values and greater affinity for CYP3A4, as compared to the parent resveratrol and its glucosides [38]. CYP3A-mediated aromatic hydroxylation and epoxidation of resveratrol is possible and results in a reactive p-benzoquinone methide metabolite that is capable of binding covalently to CYP3A4, leading to inactivation and potential drug interactions [175].

#### 5.2.2. Lignans

The lignans gomisins B and C, components of Schisandra fruit (*Schisandra chinensis*) extract, have been identified as potent inhibitors of CYP3A4* in vitro* [182]. Other evidence for the inhibitory effects of plant lignans on CYP3A4 is provided by silymarin, a mixture of flavonolignans extracted from milk thistle (*Silybum marianum*). Silymarin (0.1 mM and 0.25 mM) significantly reduced the activity of CYP3A4 in human hepatocyte cultures by 50 and 100%, respectively, as determined by the formation of 6-*β*-hydroxy testosterone [183]. Studying the effects of selected lignans from silymarin (silybin, dehydrosilybin, silydianin and silycristin) on CYP3A4 activity as determined* in vitro* by nifedipine oxidation revealed that CYP3A4 activity is inhibited as the concentration of each flavonolignan increases. However, a slight increase in activity was also observed in the presence of low flavonolignan concentrations (0.1–1 *μ*M) [184].

#### 5.2.3. Tannins

Tannic acid, a type of hydrolysable tannin commonly found in plant foods, inhibited testosterone 6-*β*-hydroxylation (CYP3A4) in human- and rat-liver microsomes with IC_50_ values of 20.2 *μ*M and 16.8 *μ*M, respectively [185].

### 5.3. Interactions between Phenolic Acids and CYP3A4

Phenolic acids do not all belong to the polyphenols, but are commonly discussed together. The interaction of phenolic acids with CYP3A4 and their potential metabolism by the enzyme would be of high relevance as the research of the more multi-member interactions of CYP3A4, polyphenols and gut microbiota advances, due to the high antimicrobial activity of phenolic acids.

#### 5.3.1. Hydroxycinnamic Acids

Caffeic acid (3,4-dihydrocinnamic acid), which do belong to the polyphenols, is one of the most common phenolic acids found in fruits, coffee, olive oil and dietary supplements. Caffeic acid has been shown to inhibit CYP3A4 activity in human liver microsomes by noncompetitive inhibition, with an IC_50_ of 0.72 *μ*M. In addition, ester and amide analogues of caffeic acid have been found to act as competitive inhibitors, with IC_50_ values ranging from 0.31 *μ*M to 0.82 *μ*M [186].

#### 5.3.2. Hydroxybenzoic Acids

Gallic acid (3,4,5-trihydroxybenzoic acid), also a member of the polyphenols and is abundant in many beverages, for example, wine, tea, pomegranate juice and olive oil, has an inhibitory effect on androstenedione 6-*β-*hydroxylase activity* in vitro* (apparent *K*_*i*_ value 70 *μ*M), which is regarded as a marker for CYP3A enzyme activity [187]. In another study, Stupans and coworkers provided additional evidence for the inhibition of CYP3A activity by gallic acid. In that study, they showed that pre-incubation of human liver microsomes with 100 *μ*M gallic acid before the assay of androstenedione 6*-β-*hydroxylase activity significantly increased the inhibitory effects of the gallic acid. In addition, they reported that the removal of gallic acid-derived products from the incubation mixture completely restored CYP3A activity [188].

## 6. Structure-Activity Relationships

Various interactions have been demonstrated between compounds belonging to the large family of polyphenols and P450 enzymes. While members of this family share many structural and functional features, existing reports do not provide sufficient information to allow us to fully understand the rules that determine the nature of these interactions. The number of hydroxyl groups, stereostructure, molecular weight and lipophilicity all seem to have some sort of effect on individual results. Up to date, the protein data bank (PDB) contains 18 crystal structure of human CYP3A4. One of the most prominent characteristics reported was the large, highly ordered hydrophobic core of phenylalanine residues above the active site [189, 190]. A recent review concluded that the CYP3A4 active site is considerably larger than the active site of any other P450 isoform [191].

CYP3A4 substrates form hydrogen bonds with the Asn74 residue of CYP3A4. Structural requirements of CYP3A4 substrates have been suggested to include a hydrogen-bond acceptor atom located 5.5–7.8 Å from the site of metabolism and 3 Å from the oxygen molecule associated with the heme [192]. A three-dimensional pharmacophore based on 38 substrates of CYP3A4 possessed two hydrogen bond acceptors, one hydrogen bond donor, and one hydrophobic region [193]. Inhibitor pharmacophores include three hydrophobes at distances of 5.2 to 8.8 Å from a hydrogen-bond acceptor, three hydrophobes at distances of 4.2 to 7.1 Å from a hydrogen-bond acceptor and at an additional 5.2 Å from another hydrogen-bond acceptor, or one hydrophobe at a distance of 8.1 to 16.3 Å from the two furthest of three hydrogen-bond acceptors [194].

Substrates or inhibitors can bind to CYP3A4 at multiple sites due to the flexible structure of this enzyme's active site [195–197]. For example, a study of the crystal structures of human CYP3A4 in complex with two well characterized drugs, ketoconazole and erythromycin, revealed that the enzyme undergoes dramatic conformational changes upon ligand binding, with an increase in the volume of the active site of more than 80%. These structures represent two distinct open conformations of CYP3A4 because ketoconazole and erythromycin induce different types of coordinate shifts [198]. CYP3A4, like many of P450 enzymes, have large and flexible substrate binding pockets capable of accommodating large substrates or alternatively two or three smaller molecules [199]. Examples on CYP3A4 cooperativity and its non-Mechaelis-Menten kinetics are found in several studies [195, 200, 201]. However, recent studies demonstrate a very complex allosteric mechanism of P450's including overlay of a multiple substrate-binding space-filling mechanism, enzyme conformational changes induced by ligands and modulation of protein-protein interactions in the enzyme oligomers [158, 202]. Allosteric behavior includes homotropic and heterotropic activation and inhibition effects depending on thermodynamic factors as demonstrated by Denisov and Sligar. The latter suggest that “functional cooperativity” best describes P450s fold that includes remote binding sites which may serve for the allosteric regulation of equilibrium and/or kinetic functional properties, including substrate binding and product dissociation, stability of oxy-complex and autoxidation [203].

### 6.1. Quantitative Structure-Activity Relationship (QSAR)

Didziapetris and coworkers developed a structure-activity relationship model to predict the probability that a compound can inhibit human CYP3A4, based on data for more than 800 compounds from various literature sources. Their model is based on GALAS methodology, which involves QSAR (quantitative structure-activity relationship) and local similarity-based corrections. The findings of the GALAS model revealed that increasing the size of the molecule via the incorporation of hydrophobic aliphatic or aromatic residues enhances the ability of the compound to inhibit CYP3A4, while a strong acidic or basic group in the molecule reduces its inhibition potential. This model emphasizes the importance of lipophilicity and the presence of hydrophobic groups on the inhibition potency of compounds, which is consistent with the phenylalanine residues already seen at the active site [123]. An additional QSAR study based on five statistical tools identified a strong correlation between the* n*-octanol/water partition coefficient (log*P*) and the binding affinity of compounds for CYP3A4 [124]. In line with these findings, a study on the influence of lipophilicity on the interactions of hydroxystilbenes with CYP3A4 revealed that methoxy-stilbenes had lower IC_50_ values and greater affinity for CYP3A4, as compared to the parent resveratrol and its glucosides. These results support the hypothesized role of lipophilicity in the interaction of polyphenols with CYP3A4 [38]. Other QSAR analyses conducted by Lewis and coworkers rationalized the lipophilicity relationships in CYP3A4 inhibitors in terms of typical active-site interactions such as hydrogen bonding and *π*–*π* stacking, whereas the multiple binding sites in the heme environment could lead to variation in gradients [204, 205].

Mao et al. showed that the traditional QSAR model applied to one data set does not lead to predictive models that would be useful for* in silico* filtering of chemical libraries and presents a multiple pharmacophore hypothesis (MPH) that is a conceptual extension of the conventional QSAR approach. Their study was based on 2,400 marketed drugs and made use of pair-wise comparisons of IC_50_ activity values for different substrates of CYP3A4. The substrates were then characterized according to the proximal and distal binding relative. MPH provides us with structural insight into how multiple substrates of CYP3A4 may interact with the enzyme (e.g., the extent to which their binding sites may lie in close proximity to one another or even overlap) [206].

## 7. Concluding Remarks

A number of studies in recent years have highlighted the potential risk inherent in the uncontrolled use of herbal medicines concurrent with conventional therapeutic regimens and emphasized the need for regulation in this field based on a set of evaluation criteria [207–211]. We propose here that it is the polyphenols in the herbal preparations that interact with CYP3A4, modify the metabolism of xenobiotics and drugs, and consequently change the active doses of prescribed medicines and the nature of the prescribed compounds. The abundance of polyphenols in many food products, the abundance of CYP3A4 in the intestine, its broad ranges of substrates/inhibitors and cooperativity, the potential involvement of gut microbiota in polyphenol-CYP3A4 interactions and* vice versa*, the extended exposure of the intestinal enzyme to polyphenol metabolites through the enterohepatic cycle and the short-term inhibition, and long-term induction of CYP3A4 by some phenolic compounds all contribute to the interest in the polyphenol-CYP3A4 interactions and their outcomes and underscore the need for further research in this area.

## Figures and Tables

**Table 1:** Potential interactions of polyphenols with CYP3A4.

Category of polyphenols	Subcategory of polyphenols	Polyphenols in category	Interaction with CYP3A4	References
Flavonoids	Flavonols	Kaempferol, galangin	Inhibition	[87, 148, 149]
		Quercetin	Inhibition	[87, 148–150]
			Induction of CYP3A4 mRNA expression *in vivo* and in prolonged-exposure assays	[87, 151, 153]
	Flavones	Apigenin, chrysin, amentoflavone	Inhibition	[73]
		Luteolin, diosmetin	Inhibition	[156]
		Flavone, tangeretin	Activation	[156]
		*α*-Naphthoflavone	Activation	[11]
	Flavonols	EGCG, ECG	Inhibition	[73, 121, 139, 162]
	Flavanones	Naringin, naringenin	Inhibition	[73, 165]
	Isoflavones	Genestein	Inhibition	[169–171]
			Activation (modest activation in clinical trials)	[172, 173]
	Anthocyanins	Anthocyanins (and anthocyanins aglycones)	Inhibition	[174]

Nonflavonoids	Stilbenes	Resveratrol (and resveratrol derivatives)	Inhibition	[38, 175–179]
	Lignans	Gomisins (B and C)	Inhibition	[182]
		Silymarin mixture	Inhibition (with slight activation at low concentrations)	[183, 184]
	Tannins	Tannic acid	Inhibition	[185]

Phenolic acids	Hydroxycinnamic acid	Caffeic acid	Inhibition	[186]
	Hydroxybenzoic acid	Gallic acid	Inhibition	[187, 188]
